# Pulmonary lesions induced by SARS-CoV-2 infection in domestic cats

**DOI:** 10.1177/03009858211066840

**Published:** 2021-12-29

**Authors:** Olivia M. Patania, Shiho Chiba, Peter J. Halfmann, Masato Hatta, Tadashi Maemura, Kristen A. Bernard, Yoshihiro Kawaoka, LaTasha K. Crawford

**Affiliations:** 1 University of Wisconsin–Madison, Madison, WI, USA; 2 University of Tokyo, Tokyo, Japan

**Keywords:** cat, COVID-19, histopathology, immunohistochemistry, lung, SARS-CoV-2

## Abstract

Severe acute respiratory syndrome coronavirus 2 (SARS-CoV-2) is the cause of coronavirus disease 2019, which ranges from fatal disease in some to mild or subclinical in most affected individuals. Many recovered human patients report persistent respiratory signs; however, lung disease in post-acute infection is poorly understood. Our objective was to describe histologic lung lesions and viral loads following experimental SARS-CoV-2 infection in 11 cats. Microscopic evaluation at 3, 6, 10, or 28 days postinoculation (DPI) identified mild to moderate patchy interstitial pneumonia, bronchiolar epithelial damage, and occlusive histiocytic bronchiolitis. Based on immunohistochemistry, alveolar septal thickening was due to CD204-positive macrophages, fewer B and T lymphocytes, type II pneumocytes, and capillary proliferation with a relative dearth of fibrosis. In blood vessel endothelium, there was reactive hypertrophy or vacuolar degeneration and increased MHC II expression at all time points. Unexpectedly, one cat from the 28 DPI group had severe subacute regionally extensive lymphohistiocytic pneumonia with multifocal consolidation, vasculitis, and alveolar fibrin. Reverse transcriptase-quantitative polymerase chain reaction identified SARS-CoV-2 RNA within the lung at 3 and 6 DPI, and viral RNA was below the limit of detection at 10 and 28 DPI, suggesting that pulmonary lesions persist beyond detection of viral RNA. These findings clarify our comparative understanding of disease induced by SARS-CoV-2 and suggest that cats can serve as an informative model to study post-acute pulmonary sequelae.

Severe acute respiratory syndrome coronavirus 2 (SARS-CoV-2) is a highly pathogenic betacoronavirus responsible for the coronavirus disease 2019 (COVID-19) pandemic.^11^ SARS-CoV-2 respiratory symptoms range from asymptomatic or mild to severe with dyspnea and/or coughing caused by pneumonia and acute respiratory distress syndrome in human patients.^30^ Many COVID-19 patients report persistent respiratory signs that last longer than the infection itself.^4^ In one study, 87% of recovered patients had persistence of at least one COVID-19-related symptom, most notably fatigue or dyspnea.^4^ In human medicine, evaluation of lung histopathology in patients with nonfatal SARS-CoV-2 infections are sparse. Findings in antemortem and postmortem lung biopsies include, but are not limited to, diffuse alveolar damage, lymphocytic to lymphohistiocytic interstitial pneumonia, congestion, pneumocyte hyperplasia, alveolar fibrinous exudates, and interstitial fibrosis.^23,25,31^ In more severe cases, hemorrhage, fibrinoid vascular necrosis, pulmonary embolism, and neutrophilic bronchopneumonia can be observed.^23,25^

Domestic cats are susceptible to SARS-CoV-2 infection and are able to transmit the virus via respiratory droplets.^3,9,12,24^ Infection in young otherwise-healthy domestic cats typically causes nonfatal respiratory disease similar to that of asymptomatic or mild SARS-CoV-2 infection in humans.^5^ The purpose of this report is to describe histopathologic findings in postmortem lung tissues from cats experimentally infected with SARS-CoV-2 that were necropsied at varying days postinoculation (DPI). To complement the cursory histopathologic findings in the respiratory tract of experimentally infected cats that we reported previously,^5^ this report provides a comprehensive histologic interpretation of the pulmonary findings concurrent with immunohistochemistry (IHC), multiplex immunofluorescence, and reverse transcriptase-quantitative polymerase chain reaction (RT-qPCR), to better characterize the cellular reactions to infection, chronic responses to tissue injury, and viral load.

## Materials and Methods

### Biosafety Statement

Research with SARS-CoV-2 was approved by the University of Wisconsin–Madison’s Institutional Biosafety Committee. This study was performed in biosafety level 3 agriculture (BSL-3Ag) laboratories at the University of Wisconsin–Madison’s Influenza Research Institute. These laboratories were approved for such use by the Centers for Disease Control and Prevention. The BSL-3Ag facility used was designed to exceed the standards outlined in Biosafety in Microbiological and Biomedical Laboratories (5th edition). Features of the BSL-3Ag facility included controlled access, entry/exit through a shower change room, effluent decontamination, negative air-pressure, double-door autoclaves, gas decontamination ports, HEPA-filtered supply and double-HEPA-filtered exhaust air, double gasketed watertight and airtight seals, and airtight dampers on all ductwork. The structure of the BSL-3Ag facility was pressure-decay tested regularly.

### Study Population

Animal studies were approved and performed in accordance with the Animal Care and Use Committee guidelines at the University of Wisconsin–Madison. The cats in this study were previously used to document persistent lung sequelae and protective immunity.^5^ Cats were assigned to 3 initial groups: group 1 (3 DPI), group 2 (6 DPI), and group 3 (10 DPI). An additional study (group 4) was later conducted to evaluate chronic infection of cats at 28 DPI. A total of 11 cats were evaluated across both studies, with 3 cats assigned to each group except for group 3 which only had 2 cats. Groups 1 to 3 and group 4 were housed separately but with similar 48% to 65% humidity at 23 °C, and with at least 15.2 air exchanges per hour. Weight and body temperature (through implanted transponders) were recorded daily. No control (mock-infected) cats were included in this study.

Eight cats were 19-week-old males obtained from the antibody profile defined/specific pathogen free (APD/SPF) breeding colony from Marshall Bioresources. Cats from this breeding colony were nonvaccinated but routinely monitored for select infectious diseases with no detectable antibody to feline herpesvirus (rhinotracheitis; FHV), feline calicivirus (FCV), feline panleukopenia virus (FPV), and feline coronavirus. Additionally, these cats were historically negative for feline immunodeficiency virus (FIV), *Chlamydia felis*, and *Toxoplasma gondii*. Six weeks prior to virus inoculation, pooled fecal samples tested positive for coccidia, and all cats were subsequently treated with oral amprolium, ALBON, and feline Fortiflora. One cat from the 10 DPI group also received an additional ALBON treatment 1 day prior and for the first 4 days of the study for empirical treatment of diarrhea even though a fecal sample 7 days prior was negative for coccidia and other parasites. Negative fecal samples were confirmed prior to viral inoculation in all cats.

Two cats in the 28 DPI group were obtained from a closed SPF (FHV, FCV, FIV, and feline leukemia virus [FeLV]) research breeding colony maintained at the University of Wisconsin–Madison. All cats in this colony were routinely tested by enzyme-linked immunosorbent assays and PCR for diseases including FeLV and FIV. The cats were 24-week-old, wildtype, male littermates. Vaccination with a combination killed virus vaccine (Elanco US, Inc) against FHV, FCV, FPV, and *Chlamydia Psittaci* was administered in the right forelimb subcutis approximately 3 months prior to the study. Mycoplasmosis, chlamydiosis, and feline infectious peritonitis were historically not reported within the colony. No clinical signs of disease were reported in these cats.

### Experimental Inoculation With SARS-CoV-2

Anesthesia and analgesia were achieved with ketamine and dexmedetomidine. Cats were inoculated with 5.2 × 10^5^ plaque forming units of SARS-CoV-2 isolate UT-NCGM02/Human/2020/Tokyo, which was passaged twice on VeroE6 cells. All cats were inoculated with a combination of routes including intraocular (50 µL per eye), intranasal (100 µL per nare), tracheal (500 µL), and orally (500 µL). Anesthesia was reversed with atipamezole.

### Postmortem Examination

A combination of ketamine and dexmedetomidine was used to reach a deep plane of anesthesia in all cats prior to euthanasia by exsanguination from the cervical arteries. Tissues were collected using standard BSL-3 biocontainment and an abbreviated sampling protocol. Macroscopic pulmonary lesions were not reported by the study prosector. The lungs were infused with 10% formalin phosphate buffer solution via the trachea before immersion into formalin. Representative sections from each lung lobe were sampled for histology, all of which included at least one grossly visible bronchus.

### Histology

Microscopic examination of tissues was performed by 1 of 2 board-certified veterinary pathologists. Tissues were submitted to the Wisconsin Veterinary Diagnostic Laboratory (WVDL) for paraffin embedding and sectioning. All formalin-fixed, paraffin-embedded (FFPE) tissues were sectioned at 5 µm and mounted on slides that were routinely stained with hematoxylin and eosin (HE). Special stains were applied to selected slides including Gomori’s trichrome, periodic acid–Schiff (PAS), and Gram stain. After initial evaluation of the range of lesions, lung sections were scored by a pathologist (LKC) who was blinded to the identity of the animals and experimental group during scoring. The sections of lung were scored as described previously.^5^ In brief, scoring was based on a 0 to 5 scoring system where 0 = absent and 5 = severe or fulminating, and the scores were averaged across lung sections for each animal, then averaged across each experimental group.

### Immunohistochemistry

Chromogen-based IHC was performed by the University of Wisconsin–Madison School of Veterinary Medicine (UW-M SVM) histology lab. FFPE representative samples of lung were sectioned at 3 to 4 µm then dried at 60 °C for 1 hour. The tissues were deparaffinized in xylene and rehydrated through a graded series of ethanol to de-ionized water. Antigen retrieval was performed using Lab Vision PT Module (Thermo Fisher Scientific; cat. no. A80400012) using pH 6.0 citrate buffer (Thermo Fisher Scientific; cat. no. TA-050-CBX) for 20 minutes and then cooled for 10 minutes. Slides were rinsed with de-ionized water and placed into a buffer solution: phosphate-buffered saline with Tween 20 (Thermo Fisher Scientific; cat. no. TA-999-PT). The subsequent steps were performed using Biocare IntelliPATH FLX automated stainer (Biocare Medical). Endogenous peroxidase was blocked using 3% hydrogen peroxide (Thermo Fisher Scientific; cat. no. 19-027033) for 10 minutes, rinsed with tap water, then rinsed in buffer. Ultra V block (Thermo Fisher Scientific; cat. no. TA-125-UB) was applied for 5 minutes and then rinsed in buffer; the latter was repeated after each sequential step on the automated stainer. The following primary antibodies were diluted using Antibody Diluent OP Quanto (Thermo Fisher Scientific; cat. no. TA-125-ADQ): (1) CD3 (DAKO; cat. no. M725401-2; 1:200 dilution), (2) CD20 (Thermo Fisher Scientific; cat. no. RB9013P; 1:400 dilution), (3) CD204 (Thermo Fisher Scientific; cat. no. 89-110-584; 1:400 dilution), (4) cytokeratin AE1/AE3 (DAKO; cat. no. M3515; 1:200 dilution), (5) factor VIII-ra (DAKO; cat. no. A008229-5; 1:1000 dilution), and (6) MHC II HLA-DR (TAL 1B5; Santa Cruz Biotechnology, Inc; cat. no. sc-53319; 1:400 dilution). The primary antibody was incubated for 30 minutes, followed by a Po-Link2 Plus Antibody Enhancer (Thermo Fisher Scientific; cat. no. NC0444899) for 10 minutes. A Po-Link2 Plus HRP Polymer (Thermo Fisher Scientific; cat. no. NC0444899) was applied for 15 minutes. 3,3′-Diaminobenzidine (DAB) solutions kit (VWR; cat. no. 95041-478) was applied for 5 minutes. Slides were removed from the automated stainer and rinsed with tap water. Slides were counterstained with hematoxylin for 5 minutes, rinsed, dehydrated, and then coverslipped. Negative controls were run by omitting the primary antibody and substituting phosphate-buffered saline.

### Multiplex Immunofluorescence

Slide-mounted FFPE sections were deparaffinized and subjected to heat-induced antigen retrieval in pH 6 citrate buffer for 20 minutes in a pressure cooker and then allowed to cool slowly to room temperature over the course of 20 minutes. Sections were then washed in 0.1% triton-X in phosphate-buffered saline (PBST) and then were blocked with 5% normal donkey serum in 0.3% triton-X in PBS blocking solution. The following primary antibodies were diluted in blocking solution and applied to sections overnight at room temperature: rabbit polyclonal SARS nucleocapsid protein (Novus Biologicals; cat. no. NB100-56576; 1:500 dilution), goat polyclonal Iba-1 (Novus Biologicals; cat. no. NB100-1028; 1:200 dilution). Negative control slides with omission of primary antibody were processed in parallel. After additional PBST buffer washes, secondary antibodies were diluted 1:500 in blocking solution and incubated for 2 hours at room temperature. Antibodies used included some combination of the following: donkey anti-rabbit Alexa 647 (ThermoFisher Scientific; cat. no. A31573), donkey anti-rabbit Cy3 (Jackson ImmunoResearch Laboratories, Inc; cat. no. 711-165-152), donkey anti-goat 647 (Jackson ImmunoResearch Laboratories, Inc; cat. no. 705-605-003), donkey anti-mouse 488 (ThermoFisher Scientific; cat. no. A21202). Washes in PBS were followed by 5 minutes of quenching of tissue autofluorescence with TrueVIEW quenching kit (Vector Labs; cat. no. SP-8400-15) followed by final washes in PBS and coverslipping with Fluoromount G mounting media (ThermoFisher Scientific; cat. no. 00-4958-02). Single Z-plane epifluorescent images were acquired on Thunder 3D Tissue microscope (Leica Microsystems) using LAS X software (Leica Microsystems). Images were collected sequentially in the Y5 (647), Y3 (546), GFP (488), and then DAPI (405) channels. Fluorescence present in multiple fluorescent channels were interpreted as autofluorescence (eg, red blood cells, tissue artifacts). Because tissue autofluorescence is minimal in the Y5 far red channel, this channel was included to improve the specificity of the fluorescent labeling. True fluorescence was interpreted as signal above the background noted during comparison to negative control slides wherein staining was completed with omission of the primary antibody. Adjustments of fluorescent images were completed using LAS X software included cropping, application of pseudocolor, and linear adjustment of the image histogram.

### RNA Extraction and RT-qPCR

Two 20-µm-thick scrolls of FFPE lung tissue from each cat were sectioned by WVDL wherein each scroll contained one section of lung tissue with a surface area ranging from 64 to 200 mm^2^. Tissue scrolls were deparaffinized with Deparaffinization Solution (QIAGEN; cat. no. 19093) according to the manufacturer’s protocol, and RNA extraction was performed using the RNeasy FFPE Kit (QIAGEN; cat. no. 73504) per the manufacturer’s instruction, resulting in a final volume of 30 µL RNA. The RT-qPCR assays were run in duplicate wells with 5 µL of sample RNA in a 25 µL reaction using TaqMan RNA-to-CT 1-Step Kit (Thermo Fisher Scientific; cat. no. 4392938). Primers and probe for the reaction were used as recommended for the 2019-nCoV RUO Kit (Integrated DNA Technologies; cat. no. 1006713), which contains the N1 SARS-CoV-2 (2019-nCoV) probe and primer mix. The 2019-nCoV_N_Positive Control (Integrated DNA Technologies; cat. no. 10006625) DNA plasmid was used to develop a standard curve for quantitation. An endogenous control gene, feline hydroxymethylbilane synthase (HMBS), was previously described^14^ and was used to validate RNA integrity within the FFPE samples: 100 µm of forward primer (TGGCAGTGCTGAAAGCCTTA), 100 µm of reverse primer (TTAGAGAGCGCAGTATCAAGAATCTT), and 25 µm of probe (FAM-TTGAAATCGTTGCTATGTCCACCACAGG-TAMSp) (Integrated DNA Technologies). Nontemplate controls for both SARS-CoV-2 and the endogenous control gene were included for each assay. All RNA samples were positive for the endogenous control, and all nontemplate controls were negative. Cycling conditions were modified to 30 minutes at 48 °C, 10 minutes at 95 °C followed by 40 cycles of 15 seconds at 95 °C and 60 seconds at 55 °C using an Applied Biosystems StepOnePlus Real-Time PCR System (Thermo Fisher Scientific; cat. no. 4376600). A positive Ct cutoff of 37 cycles was used with a limit of detection of 0.4 nucleoprotein (N) gene copies per microliter of control DNA plasmid.

## Results

### Clinical Findings

After inoculation, all cats were asymptomatic. Temperatures, which were recorded daily, remained stable and within normal range. Body weight gradually increased throughout the course of the experiment as expected for animals of this age.

### Histopathology and Immunohistochemistry

**Figures 1–4.**
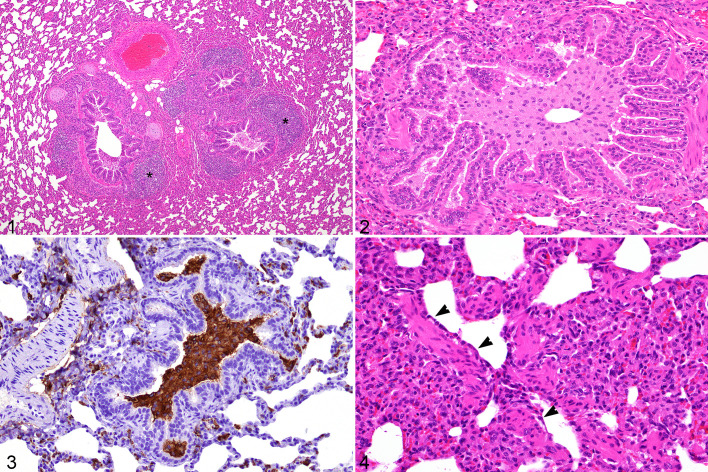
Bronchial and bronchiolar lesions, SARS-CoV-2 infection, lung, cat, 3 days postinoculation. **Figure 1.** Hyperplasia of bronchus-associated lymphoid tissue (*). Hematoxylin and eosin (HE). **Figure 2.** Bronchiole with near-complete occlusion of the lumen by epithelioid macrophages. HE. **Figure 3.** Bronchiolar lumina are occluded by epithelioid macrophages with strong cytoplasmic immunolabeling for CD204. **Figure 4.** Terminal bronchiole with attenuation or loss of epithelium (arrowheads) and focal narrowing of the lumen. HE.

Evaluation of the lung revealed lesions of the bronchi, bronchioles, interstitium, and vasculature, with the bronchioles being the most severely and diffusely affected as detailed below. Within occasional bronchi, bronchial glands were infiltrated or surrounded by a minimal to mild amount of inflammation composed of lymphocytes and macrophages with fewer plasma cells and neutrophils. Changes that were prominent at later time points included moderate hyperplasia of the epithelium with layering of nuclei and multifocal herniation of epithelium below the smooth muscle layer. Leukocytes occasionally migrated into the epithelium. Bronchial lumina infrequently contained sloughed epithelial cells or mucus filling less than 25% of the lumen. Hyperplasia of bronchus-associated lymphoid tissue (BALT) was mild to moderate (Fig. 1) and composed of a mixture of CD3- and CD20-positive lymphocytes, as confirmed by IHC labeling. As reported previously, bronchitis and lymphoid hyperplasia appeared less prominent at later time points.^5^

In addition to histiocytic bronchiolitis with luminal occlusion reported previously,^5^ the bronchioles and terminal bronchioles exhibited additional changes. Bronchiolitis was characterized by partial to approximately 99% complete occlusion of bronchiolar lumina by plugs consisting of round to epithelioid cells with foamy to slightly fibrillar eosinophilic cytoplasm consistent with viable and degenerative macrophages.^5^ Severe occlusion of bronchioles was found even in otherwise-unaffected areas with no alveolar septal thickening or inflammation (Fig. 2). By IHC, the cellular infiltrate had strong cytoplasmic labeling for Iba-1 and CD204 (macrophages; Fig. 3). At all examined time points, labeled macrophages were adhered to the bronchiolar epithelium, forming small aggregates in the interstitium adjacent to bronchioles, and randomly distributed within thickened alveolar septa. Immunolabeled macrophages appeared more numerous with Iba-1 labeling than with CD204. At later time points, increased numbers of bronchioles showed variable remodeling with occasional erosion, robust epithelial proliferation with multifocal mild multilayering of nuclei, moderately frequent herniation of epithelium below the muscle layer, and segmental bronchiolar constriction particularly in regions with adherent histiocytic luminal exudate. There was minimal peribronchiolar fibrosis observed, particularly in areas with severe bronchiolar occlusion. Overlying the smooth muscle of the terminal bronchioles, the columnar epithelium was frequently replaced with attenuated to eroded epithelium with occasional areas of apparent adherence between denuded regions (Fig. 4). There was mild, variable smooth muscle hypertrophy of terminal bronchioles and bronchioles noted on HE and trichrome stains at later time points (data not shown).

**Figures 5–13.**
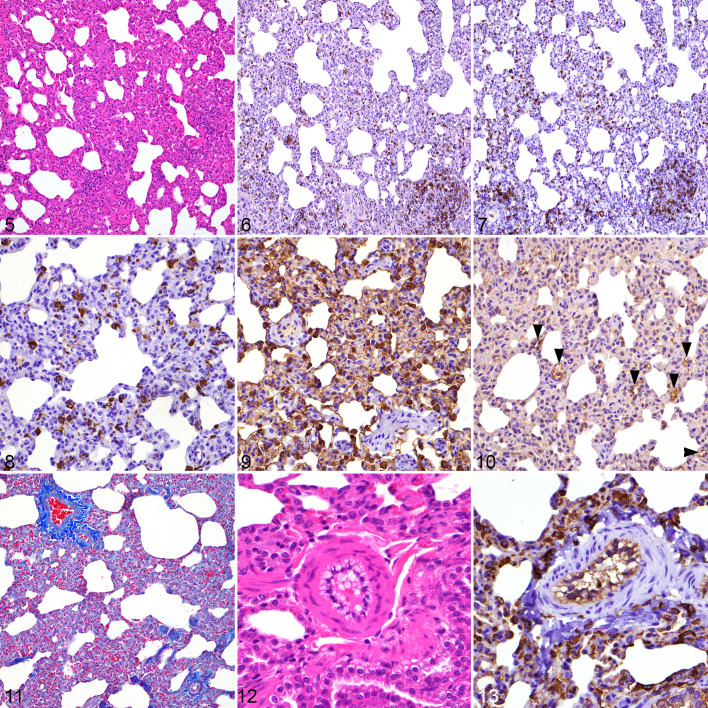
Interstitial lesions, SARS-CoV-2 infection, lung, cat. **Figure 5.** Moderate thickening of alveolar septa; 28 days postinoculation (DPI). Hematoxylin and eosin (HE). **Figure 6.** Scattered B lymphocytes with strong immunolabeling for CD20 contribute to septal thickening; 28 DPI. **Figure 7.** T lymphocytes with strong immunolabeling for CD3 form small aggregates in thickened alveolar septa; 28 DPI. **Figure 8.** Macrophages with strong immunolabeling for CD204 are present within alveolar septa; 10 DPI. **Figure 9.** Increased numbers of type II pneumocytes with strong cytoplasmic immunolabeling for cytokeratin; 10 DPI. **Figure 10.** Endothelial cells and nascent blood vessels (arrowheads) with moderate cytoplasmic immunolabeling for FVIII-ra within the septa; 10 DPI. **Figure 11.** Lack of mature collagen in thickened alveolar septa; 6 DPI. Gomori’s trichrome stain. **Figure 12.** Endothelial cells around blood vessels are reactive with vacuolar degeneration; 10 DPI. HE. **Figure 13.** Endothelial cells lining blood vessels show strong immunolabeling for MHC II. There is also strong diffuse immunolabeling of type II pneumocytes as well as bronchiolar epithelium; 10 DPI.

Interstitial findings that were previously reported included patchy to coalescing thickening of the alveolar septa with perivascular lymphoid nodules.^5^ Patchy to regionally extensive thickening of alveolar septa was sometimes up to 10× normal thickness and varied across lung lobes for any given animal (Fig. 5). In early time points, inflammation was more severe and the interstitium was more severely thickened, while at later time points inflammation was greatly reduced yet the interstitium remained thickened with a notable lack of mature collagen deposition based on trichome stain.^5^ Inflammatory cells were noted on HE multifocally within the alveolar septa and formed multifocal perivascular and random aggregates. Thickening of the interstitium was suspected to be due to a mixture of inflammatory cells, type II pneumocytes, and possible endothelial cells.^5^

Further investigation with IHC was pursued to determine the specific cell types that contributed to the interstitial thickening. The inflammatory infiltrate was composed of cells exhibiting strong CD20 immunolabeling (B lymphocytes; Fig. 6), cells exhibiting strong CD3 immunolabeling (T lymphocytes; Fig. 7), and CD204-positive cells (macrophages; Fig. 8) with fewer plasma cells identified by HE. IHC for major compatibility complex II (MHC II) demonstrated strong cytoplasmic immunolabeling of abundant cells within perivascular lymphoid aggregates, thickened interstitium, and bronchiolar lumens consistent with B cells, T cells, and macrophages. There were scattered neutrophils admixed with the interstitial inflammatory infiltrate at all time points, most clearly identified by nuclear morphology seen on nuclear counterstains of negative control IHC slides (data not shown). IHC for cytokeratin, a marker for epithelial cells, revealed increased numbers of type II pneumocytes scattered within alveolar septa with strong cytoplasmic immunolabeling (Fig. 9). Additionally, labeling for factor VIII–related antigen (FVIII-ra), an endothelial cell marker, revealed minimal to sometimes moderately increased labeling within thickened alveolar septa, suggestive of endothelial hyperplasia or hypertrophy. Endothelial cells exhibited moderate to strong positive cytoplasmic immunoreactivity. At later time points increased numbers of capillaries and small caliber vessels lined by endothelial cells strongly labeled with FVIII-ra were seen within the thickened septa (Fig. 10). Additionally, at later time points, abnormal capillary proliferations in some areas appeared progressive and disorganized, resembling pulmonary capillary hemangiomatosis. Fibrosis was overall minimal within most lobes, even at later time points (10 and 28 DPI), having a notable lack of mature collagen in markedly thickened alveolar septa and constricted bronchioles, which was confirmed via Gomori’s trichrome stain (Fig. 11). Unlike bronchioles, alveolar spaces were mostly clear with only minimal to mild, multifocal, intra-alveolar fluid accumulation in some lobes throughout all time points. Hemorrhage was rarely observed, but when present typically affected only one lobe in at least one cat from each group, except for one cat from the 28 DPI group with fulminant pneumonia that had hemorrhage in nearly all lobes. There was patchy atelectasis that was overall minimal to mild across various time points, sometimes with adjacent distended alveoli as previously reported.^5^

Reactive endothelial cells and/or damage to the vascular endothelium was observed at all time points in nearly all lung lobes.^5^ Endothelial changes ranged from plump reactive endothelial cells to marked cytoplasmic vacuolar degeneration (Fig. 12). MHC II staining performed to identify inflammatory cells within the lung also showed strong cytoplasmic immunolabeling of the endothelium of medium caliber vessels, particularly in affected regions of the lung (Fig. 13), while other vessels in lesser affected regions of the lung demonstrated faint to minimal cytoplasmic labeling. MHC II also showed faint to moderate cytoplasmic immunolabeling of bronchiolar but not bronchial epithelial cells (data not shown). Perivascular expansion of adventitial connective tissue by increased white space or proteinaceous fluid (perivascular edema) was least apparent in cats from the 3 DPI group and was minimal to moderate in the 28 DPI group. Megakaryocytes were observed within presumed interstitial capillaries at all time points and were most numerous in cats from the 3 DPI group with an average of 5 to 10 cells observed in ten 200× fields and a maximum of 14 megakaryocytes in ten 200× fields in a given lobe. The majority of cats at later time points averaged 1 to 5 megakaryocytes within ten 200× fields, and numbers decreased over time.

**Figures 14–20.**
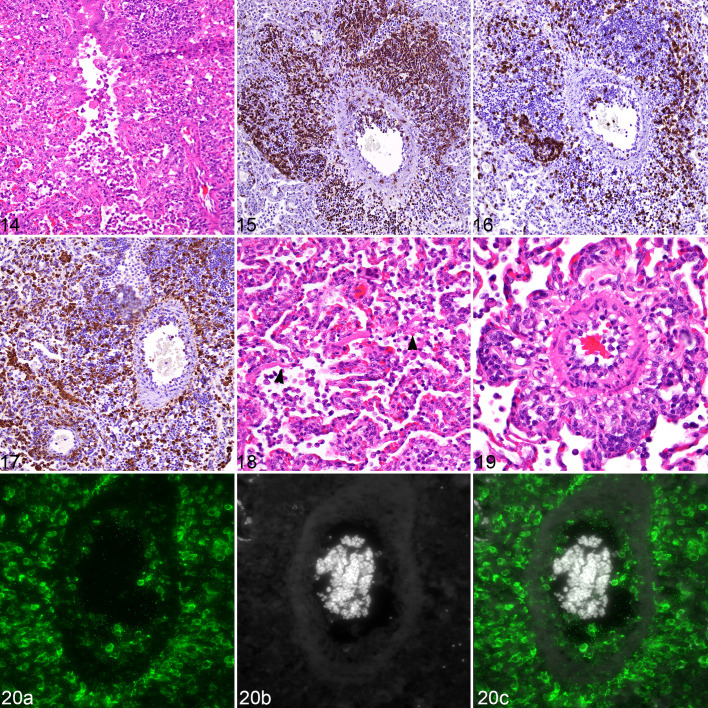
Fulminant lesions, SARS-CoV-2 infection, lung, cat, 28 days postinoculation. **Figure 14.** Bronchiolar epithelial cell ulceration and necrosis with atelectasis and consolidation due to inflammatory exudate in bronchiolar lumen and alveoli. Hematoxylin and eosin (HE). **Figure 15.** B lymphocytes with strong immunolabeling for CD20 predominate in perivascular lymphoid aggregates, are noted within the wall of an artery, and extend into the adjacent alveolar septa and perivascular connective tissue. **Figure 16.** T lymphocytes with strong immunolabeling for CD3 are found in lymphoid aggregates, perivascular connective tissue, alveolar septa, and within the wall of an artery. **Figure 17.** Macrophages with strong immunolabeling for CD204 are scattered within lymphoid aggregates, infiltrate alveolar septa, partially fill alveoli, and are noted within the vessel wall of an artery. **Figure 18.** Collapsed alveoli contain increased numbers of mixed inflammatory cells as well as fibrin (arrowheads). HE. **Figure 19.** Moderately severe vasculitis with reactive and mildly vacuolated endothelial cells and mild perivascular infiltrate. HE. **Figure 20.** Immunofluorescence labeling for Iba-1 (macrophages). (a) Iba-1 was pseudocolored green during image acquisition with a far-red light source. (b) A grayscale image of autofluorescence acquired with a GFP light source shows autofluorescent red blood cells in the vessel lumen along with tissue detail including the vessel wall. (c) The merged image shows that macrophages with strong labeling for Iba-1 surround and infiltrate the wall of an artery and cluster at the luminal surface in apparent association with endothelium.

One of three 28 DPI cats had fulminant lung lesions with severe vasculitis, mixed perivascular, interstitial, and alveolar inflammation, and alveolar fibrin accumulation, as previously reported.^5^ Bronchial and bronchiolar epithelial cell ulceration and necrosis was multifocal with fibrin accumulation near areas of necrosis (Fig. 14). Multiple lung lobes had regionally extensive severe inflammation forming coalescing perivascular lymphoid aggregates of CD20- and CD3-positive lymphocytes (Figs. 15 and 16, respectively) and CD204-positive macrophages (Fig. 17) within the interstitium. Alveoli were often collapsed or consolidated and contained mixed inflammatory infiltrates (Fig. 18), including macrophages, neutrophils, and rare eosinophils. In these areas, alveoli contained cellular debris along with a mild amount of homogeneous eosinophilic fibrillar PAS-positive material consistent with fibrin, as previously reported.^5^ Vasculitis affecting medium-caliber vessels was present in the most severely inflamed areas with macrophages, B cells, and T cells present within vascular walls and associated with endothelium that was hypertrophied with multifocal vacuolar degeneration (Figs. 15–17, 19, and 20). Rare multifocal fibrin microthrombi were observed in the capillaries of 2 lobes. Moderate to severe endothelial reactivity and damage was present in most lung lobes. Perivascular edema was moderate. Hemorrhage was more extensive than in other cats, with nearly all lobes having minimal to moderate coalescing areas of hemorrhage filling alveoli. Infectious organisms were not observed using HE, PAS, and Gram staining within the lungs and nasal cavity.

### Immunofluorescence for Viral Antigen and Iba-1

The ability for a coronavirus to infect and survive within macrophages is a crucial step in the pathogenesis of other coronavirus-mediated diseases in cats, including feline infectious peritonitis caused by the mutated coronavirus FIPV.^15^ Given the presence of chronic histiocytic infiltrates within bronchiolar lumens, increased interstitial macrophages in the lungs of all infected cats, and the macrophage component of vasculitis seen in an individual cat, we sought to determine whether virus could be detected within macrophages in cats infected with SARS-CoV-2. Multiplex immunofluorescent IHC was performed to detect the presence of viral antigen (nucleocapsid viral antigen antibody) and macrophages (Iba-1 antibody) in representative lung sections of cats at 3, 10, and 28 DPI. At 3 DPI, within rare bronchial lumens, sparse viral antigen was found on the superficial surface of bronchial epithelium and admixed with necrotic cellular debris and sloughed epithelia.^5^ Viral antigen was not detected at the other time points. Labeling with Iba-1 revealed abundant macrophages within the interstitium and lumen of bronchioles; this was similar to the distribution of CD204-positive macrophages, though Iba-1-postive macrophages were more numerous. No colocalization between viral antigen and macrophages was noted in any of the examined sections (data not shown). Iba-1 labeling of representative lung sections from 3, 10, and 28 DPI demonstrated similar distribution of abundant macrophages, and in the one cat from the 28 DPI group with fulminant pneumonia, macrophages were noted within occasional medium-caliber blood vessel walls particularly concentrated at the endothelium (Fig. 20).

### Quantitation of SARS-CoV-2 RNA in Lung

**Figure 21.**
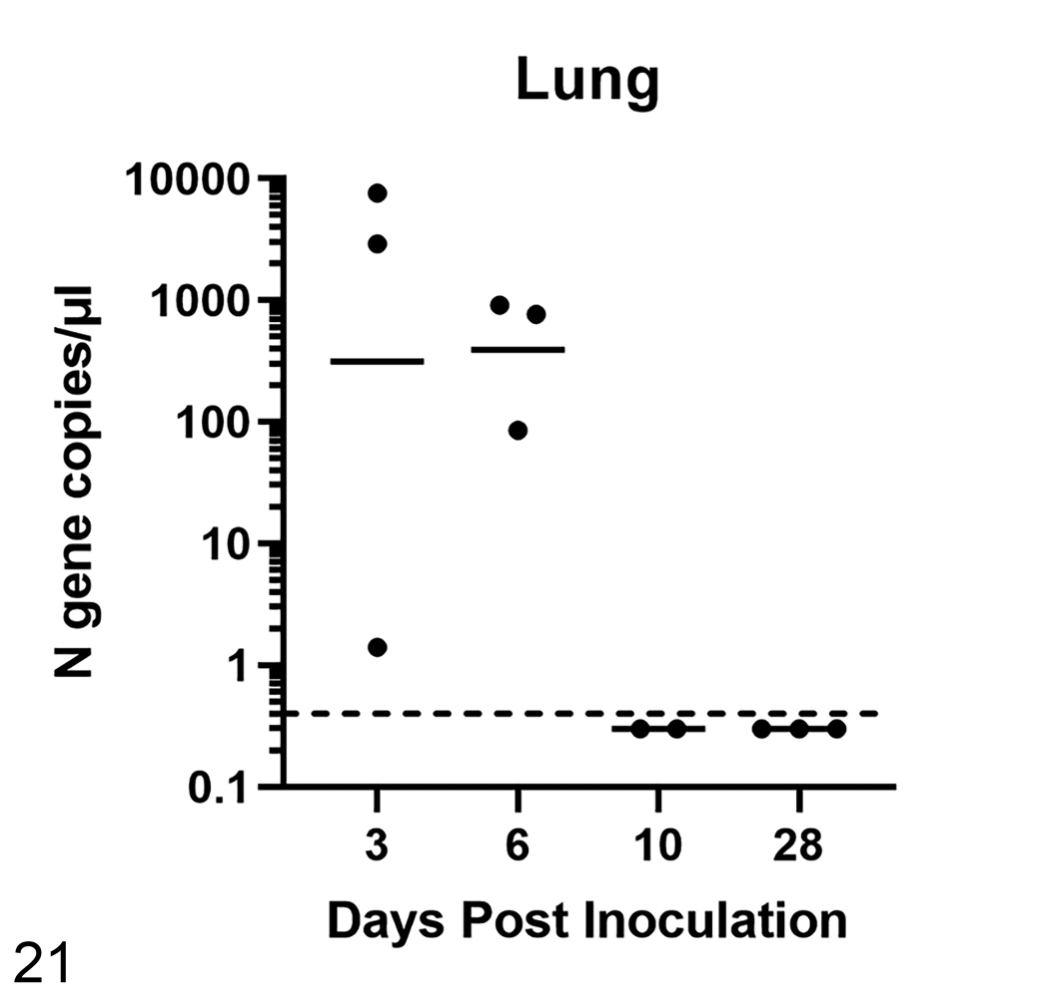
Viral load in the lung of SARS-CoV-2 infected cats. Viral RNA was detected by RT-qPCR at 3 and 6 days postinoculation (DPI). The highest viral loads were present at 3 DPI. Values were below the limit of detection at 10 and 28 DPI and are considered negative. The solid horizontal lines represent geometric means for each group. The dotted horizontal line indicates the limits of detection at 0.4 nucleoprotein (N) gene copies per microliter of RNA.

All cats from the 3 and 6 DPI groups had detectable SARS-CoV-2 RNA in FFPE sections of lung by RT-qPCR (Fig. 21). The highest number of N gene copies of viral RNA was found in 2 of the 3 cats from the 3 DPI group. However, viral RNA values were below the limit of detection in all cats from the 10 and 28 DPI groups.

## Discussion

Histologic examination of the lungs showed chronic lesions that persisted beyond detection of viral SARS-CoV-2 RNA. Expanding on previously reported findings,^5^ we found that major pulmonary findings, including hypertrophy and vacuolation of vascular endothelium, thickening of alveolar septa, bronchiolar epithelial damage, remodeling of terminal bronchioles, and occlusive histiocytic bronchiolitis (bronchointerstitial pneumonia). Although there was a strong persistent histiocytic component associated with the broncholitis and interstitial thickening (and vasculitis in one case), we did not see evidence of any viral antigen within macrophages. This distinguishes SARS-CoV-2 from feline infectious peritonitis virus, a coronavirus whose pathogenesis relies on viral persistence within infected macrophages/monocytes.^16^ SARS-CoV-2 antigen was found within the sloughed epithelium in the bronchial lumens and along the superficial surface of bronchial epithelium as previously reported.^5^ Because the bronchi were not as severely affected as the bronchioles and alveoli, it is likely that this represents shedding of infected cells and that a time point earlier than 3 DPI may be better suited to detect SARS-CoV-2 infection of cells distal to the bronchi.

We further investigated the cell types causing the interstitial thickening by performing IHC. The interstitium was thickened by a mixture of abundant Iba-1- and CD204-positive macrophages, scattered B and T lymphocytes, and mild type II pneumocyte hyperplasia. Type II pneumocyte hyperplasia was only evident using cytokeratin immunolabeling, as increased numbers of pneumocytes were multifocal and scattered, and thus did not form characteristic columns of tombstone-like cells lining the alveoli. Both B and T cells were scattered within the interstitium and found in perivascular lymphoid nodules. A significant component of the interstitial infiltrate and bronchiolar plugs were positive for Iba-1 and CD204. Iba-1 is considered to be a monocyte/macrophage lineage marker, can be used as a pan-macrophage marker, and also labels dendritic cells in a range of histiocytic diseases in cats.^20^ In contrast, CD204, which showed less abundant labeling than Iba-1 in these cases, has been documented in M2 macrophages, alveolar macrophages of the feline lung, and interstitial dendritic cells in some histiocytic diseases.^26^ M2 macrophages are typically associated with anti-inflammatory, wound healing pathways, and Th2 immune responses like angiogenesis^13,26^ and have been reported in other animal models of SARS-CoV-2.^7,8^ Activated alveolar macrophages and cytotoxic T cells have been reported to play a role in COVID-19 disease human patients as well,^6,19,28^ which highlights pathways that can be explored in future studies.

Interestingly, in addition to the inflammatory infiltrate, the interstitium was thickened by persistent, if not progressive, proliferation of spindle-shaped to slightly plump cells that were independent of interstitial fibrosis. Together with the 28 DPI finding that the interstitium contained an increased density of small-caliber blood vessels within the thickened interstitium, it is very likely that capillary proliferation is a component of the interstitial thickening seen in early time points. The increased FVIII-ra labeling within the thickened interstitium supports this interpretation. In fact, vascular changes such as angiogenesis, endothelial cell infection, vasculitis centered on the endothelium (endothelialitis), and thrombosis have been reported as key components of the lung lesions in human patients that die from COVID-19.^1,27^ In later time points, vascular proliferation within the interstitium appeared progressive and disordered in patchy areas that resembled pulmonary capillary hemangiomatosis, a condition defined by abnormal capillary proliferation that can contribute to pulmonary hypertension and is thought to develop due to neoangiogenesis secondary to injuries to pulmonary vasculature.^18,29^ The endothelium was reactive, hypertrophied, and even vacuolated across all time points evaluated in this study, which was suggestive of endothelial injury. The endothelium also demonstrated increased expression of MHC II in multifocal vessels of various calibers, particularly in affected regions of the lung. Upregulation of MHC II expression in pulmonary endothelium is a change induced by TNF-α that is seen in inflammatory conditions including FIP.^2,15^ MHC II expression was also noted in bronchiolar epithelium but not bronchial epithelium, an interesting pattern given the predilection of lesions in this study centered on endothelium and bronchiolar epithelium yet seemed to spare bronchial epithelium.

Several other lesions resembled those reported in fatal SARS-CoV-2 infections in humans, with a few distinctions. In a series of human autopsy cases, increased platelet-rich thrombi and megakaryocytes were observed in the pulmonary microvasculature of SARS-CoV-2 patients compared with those that died from acute respiratory distress syndrome, suggesting that megakaryocytes play a role in the prothrombotic events associated with SARS-CoV-2 infection.^21^ Megakaryocytes within presumed interstitial capillaries were reported previously in these cats at all time points with higher numbers observed in cats from the 3 DPI group.^5^ Rare fibrin microthrombi were observed in only one cat from the 28 DPI group for which increased megakaryocytes were not observed. The paucity of thrombi in the lungs of cats, which is an important underlying mechanism for multi-organ failure, hypoxia, and death in humans, is suggestive of a species-specific difference in the host immune response to SARS-CoV-2 rather than a consistent feature induced by the pathogen itself. Hyaline membrane formation was not a significant finding in the majority of our cats, as has been shown by others.^9^ However, one more severely affected cat did have fibrin present within alveolar spaces. The lack of membrane formation, which is characteristic in severe human cases, may also suggest that hyaline membrane formation is the result of the host response to the pathogen. In fact, hyaline membranes are inconsistent features in several susceptible animal models that exhibit SARS-CoV-2-induced clinical signs and moderate to robust lung lesions including Syrian hamsters and nonhuman primates.^10,17,22^ Alternatively, it may reflect subtle species differences in cellular tropism for the virus. Thickening of alveoli septa and histiocytic bronchiolitis that we observed in cats have been observed in postmortem samples in humans.^23,25^ Thickened septa can contribute to poor oxygen exchange and partial to complete plugging of small airways can hinder airflow and disrupt mucociliary clearance. One 28 DPI cat had severe subacute pulmonary disease with vasculitis, similar to what is reported in humans that die from COVID-19.^1,23^ The underlying mechanisms for the lesions in this cat were unclear but may be similar to otherwise young healthy humans who develop severe COVID-19 disease with no underlying cause for the extreme severity.

SARS-CoV-2 RNA was demonstrated in FFPE lung tissue by RT-qPCR at 3 and 6 DPI but values were below the limit of detection in cats from the 10 and 28 DPI groups. We previously reported that no virus was detected by viral plaque assay in nasal or tracheal swabs after 6 DPI in these cats, suggestive of viral clearance from respiratory tissues by 10 DPI and certainly by 28 DPI.^5,12^ Therefore, we would not expect the virus to persist in the lungs at 28 days after infection. These findings may suggest that cytokine-mediated damage outlasts the infection and is particularly severe in some individuals. An alternative interpretation, that lung lesions in these cats may reflect increased susceptibility to other necrotizing pathogens, is less likely as these cats were specific pathogen–free research cats housed in a clean facility. There was no overt evidence of bacterial, fungal, or additional viral pathogens within the lungs during histologic examination; however, specific testing for bacteria, fungi, or additional viruses was not performed in all cats. Gram and PAS staining in the lungs and nasal cavity of the cat with fulminant lung lesions did not reveal infectious organisms.

Our findings in domestic cats suggest that SARS-CoV-2 can induce long-lasting lung lesions that are independent of diffuse alveolar damage, despite a lack of clinical signs. These findings add critical comparative knowledge to our understanding of COVID-19 disease, as the vast majority of infected human patients experience subclinical to moderate, nonfatal COVID-19 without an exaggerated immune response or so-called “cytokine storm.” Although these cats did not have clinical signs, we speculate that the functional consequences of the lung lesions observed in our study could potentially include ventilation/perfusion mismatch and decreased lung function. Other potential sequelae could include exercise intolerance, exacerbation of coexisting lung lesions such as feline asthma, or susceptibility to infection due to impaired clearance of pathogens by remodeled and metaplastic bronchioles. Because of evolutionary tendencies for many felids to hide their clinical signs, overt signs of early respiratory distress may not be readily detected in domestic cats, requiring more careful measurement of lung function in order to rule out the presence of lung disease. Lung function tests were not assessed in the cats in our study. However, our findings suggest that feline patients may be a compelling model for a subset of human COVID-19 patients that are not hospitalized or are asymptomatic for the duration of their infection. Symptomatic survivors of COVID-19 report a plethora of persistent symptoms after viral clearance, including but not limited to fatigue, dyspnea, chest pain, and cough.^4^ In some human cases these symptoms have reportedly lasted weeks to months. In our study, cats did not exhibit clinical signs during viral shedding or after viral clearance; however, given the persistence of lung lesions at 28 DPI, it remains unknown if clinical signs could develop later due to chronic long-term pulmonary disease resulting from SARS-CoV-2 infection.

One significant limitation in this study is the lack of negative control cats. The original study design did not include mock-inoculated or naïve cats; therefore, control tissues were not available to the pathologists. Despite this limitation, our study enabled the comparative assessment of lesions that progress over time following SARS-CoV-2 inoculation, providing important insight into post-acute subclinical disease. Additional studies evaluating clinical signs and disease progression in cats infected with SARS-CoV-2 and concurrent comorbidities are warranted and may provide valuable insights for practitioners and diagnosticians working with cats. Furthermore, our study provides a detailed time course of lung lesions, which in human patients is extremely challenging to investigate, and the findings in cats can provide insight into the pathogenesis of COVID-19.
